# The Moderating Role of Education on the Relationship Between Perceived Stereotype Threat and False Memory in Aging

**DOI:** 10.3389/fpsyg.2020.606249

**Published:** 2021-01-12

**Authors:** Anne-Laure Gilet, Christelle Evrard, Jean-Michel Galharret, Fabienne Colombel

**Affiliations:** ^1^ Laboratoire de Psychologie des Pays de la Loire (LPPL – EA 4638), Université de Nantes, Nantes, France; ^2^ Laboratoire de Mathématiques Jean Leray (LMJL-UMR 6629), Université de Nantes, Nantes, France

**Keywords:** memory, aging, DRM, false memories, stereotype threat

## Abstract

Studies regularly show that an age-based stereotype threat impairs older adults’ performance on memory tasks. Results regarding stereotype threat effects on false memories are less clear. Some studies suggest that education may moderate the relationship between an age-related stereotype threat and episodic memory performance in older adults. The present study aimed at examining the moderating role of education on the relationship between perceived stereotype threat (PST) and false memories in older adults. With this aim, 82 adults between 60 and 70 years of age performed a Deese-Roediger-McDermott (DRM) task followed by a free recall test and completed questionnaires assessing both their perception of an age-based stereotype threat and their education level. Regression analyses showed no effect of PST on the production of critical lures. However, as was expected, our results showed that in higher educated older adults, as the perception of stereotype increases, the production of critical lures increases. These results confirm the moderating role of education and highlight its key role in the relationship between the age-based stereotype threat and older adults’ susceptibility to false memories.

## Introduction

Age-related stereotypes are very present in our environment and our daily lives. Research shows that both young and older adults share many positive and negative perceptions and stereotypes about aging (e.g., Hummert et al., 1994; Grühn et al., 2011). However, people’s representations of older adults are more imbued with negative perceptions than those of younger adults (e.g., Kite et al., 2005; Hummert, 2011). The effects of negative age-related stereotypes on older adults’ episodic memory performance have been regularly highlighted in the literature. Several studies have shown that older participants previously subliminally exposed to negative age-related stereotypes performed worse in memory tasks than older adults exposed to positive age-related stereotypes or non-exposed to age-related stereotypes (e.g., Hess et al., 2004; Levy, 2009). In other studies, researchers observed that memory performance of participants aged 60 and over was worse (and the age-related difference increased) when the memory component of the task was emphasized than when it was not (e.g., Rahhal et al., 2001; Chasteen et al., 2005; Desrichard and Köpetz, 2005). In such laboratory conditions, older adults may perceive and experience what Steele and Aronson (1995, p. 797) called the stereotype threat, i.e., “being at risk of confirming, as self-characteristic, a negative stereotype about one’s group.”

A growing body of research has shown that an induced age-based stereotype threat impairs older adults’ performance in a large variety of cognitive tasks (e.g., Barber and Mather, 2014; Lamont et al., 2015; Barber, 2017) such as episodic memory (see Armstrong et al., 2017 for a recent meta-analysis on this topic). Several hypotheses have been proposed to explain these effects relying either on cognitive or on motivational mechanisms. According to the executive control interference hypothesis (Schmader and Johns, 2003), the stereotype threat takes up cognitive resources, thereby increasing the cognitive load placed upon the cognitive system (particularly upon working memory), which induces a performance decrease. Recent results showing a deleterious effect of an age-based stereotype threat on older adults’ associative memory (Brubaker and Naveh-Benjamin, 2018) or the use of controlled processes while strengthening the use of automatic processes (Mazerolle et al., 2012) offer support to this cognitive hypothesis. The motivational explanation is based on the regulatory focus theory (Higgins, 1997). Research suggests that when participants perform a memory task under a stereotype threat, they adopt a prevention focus, become more vigilant to errors and try to avoid committing errors as much as possible (see Barber, 2017 for an extensive presentation; Seibt and Förster, 2004; as opposed to a promotion focus leading participants to remember as many words as possible whatever the number of errors they would make). This motivational hypothesis finds support in studies showing a decrease in correct recalls and/or a decrease in memory errors (e.g., Hess et al., 2003, 2009; Barber and Mather, 2013a,b; Popham and Hess, 2015; Barber, 2017).

One type of memory errors that is likely to be affected by an age-based stereotype threat is false memory. False memories are usually defined as distorted memories of events that occurred or memories of events that did not happen (Roediger and McDermott, 1995) and are associated with high levels of certitude. One of the most widely used paradigm to study individuals’ susceptibility to false memories is the Deese-Roediger-McDermott (DRM; Deese, 1959; Roediger and McDermott, 1995) paradigm, in which participants study lists of semantically related words (e.g., bed, awake, tired, dream, etc.) strongly associated with a critical lure that is never presented (e.g., sleep), before being asked to recognize and/or recall as many words as possible without guessing. Participants often wrongly recall or recognize critical lures, thus making false memories. A large amount of studies has focused on factors influencing the occurrence of false memories. Research has shown that factors, such as age (e.g., Tun et al., 1998; Dehon and Brédart, 2004), positive and high arousing moods (e.g., Storbeck and Clore, 2005; Corson and Verrier, 2007; Storbeck and Clore, 2011), cognitive style (e.g., Corson et al., 2009), stress (Payne et al., 2002), the use of long rather than short DRM lists (e.g., Robinson and Roediger, 1997), or a relational rather than an item-specific processing during encoding (e.g., Hunt and Einstein, 1981), increase the occurrence of false memories.

To our knowledge, only three studies investigated the effects of an induced age-based related stereotype threat on older adults’ propensity to false memories (Thomas and Dubois, 2011; Wong and Gallo, 2016; Smith et al., 2017), but these studies did not yield any consensual results. In these studies, participants were first presented with DRM lists, then some of them were exposed to a blatant age-based stereotype threat induction (by reading short paragraphs on age-related cognitive decline), and finally performed a recognition task. Thomas and Dubois (2011) observed that adults aged between 60 and 74 years were more inclined to falsely recognize critical lures in the age-based stereotype condition than in the control condition (reading a short text on language processing after encoding DRM lists). No significant effects of the age-based stereotype threat were found on hits. On the contrary, Wong and Gallo (2016) found that older adults (65–87 years) exposed to an age-based stereotype threat induction made fewer false alarms on the critical lures than their counterparts in the control condition who read an age-neutral text on language research. However, it is worth noting that Wong and Gallo (2016) had included a DRM warning before the participants performed the recognition task. Participants were informed that some of the words in the recognition task were strongly associated with words they had studied but that these words were not included in the initial list. Participants were then told to avoid confusion between those words and the words they had studied. As this warning may partly explain the contradictory results, and in an attempt to explain the discrepancies between Thomas and Dubois (2011) and Wong and Gallo (2016) studies, Smith et al. (2017) chose to include four experimental conditions combining the presence or the absence of an age-based stereotype threat and the presence or the absence of a DRM warning. This warning was presented before the recognition task and consisted of a short text explaining the principles of the DRM paradigm, i.e., that studied words were all related to another word that is often falsely remembered by participants. Participants aged 56–90 years thus performed a DRM associated with a recognition test in one of the four experimental conditions. Consistent with the results of Thomas and Dubois (2011), they showed that older adults exposed to negative age-related stereotypes made more false recognition of critical lures than older adults non-exposed to negative age-related stereotypes and did not replicate the results obtained in Wong and Gallo (2016). Interestingly, Smith et al. (2017) also highlighted the moderating role of several factors such as years of education and retirement status. They showed that older adults’ susceptibility to false recognitions (false memories) increases under the stereotype threat even more in retired or highly educated participants.

Armstrong et al. (2017) also investigated the potential moderator effects of education, age, and type of memory tests. In their meta-analysis comprising studies using blatant or subtle (e.g., non-direct communication of memory decline) stereotype threat manipulation, they concluded that neither the years of education nor the age moderated the association between the age-based stereotype threat and the episodic memory performance in older adults. They also showed that the age-based stereotype threat effect was significant in free recall tasks but not in cued-recall or recognition tasks. This is consistent with the idea that, in addition to aging, a stereotype threat reduces the ability to use strategic processes leading to larger memory impairments in older adults (e.g., Craik and McDowd, 1987; Mazerolle et al., 2012).

The objective of the present study was to investigate the moderator role of years of education on the relationship between the perceived age-based stereotype threat and the production of false memories in older adults. Unlike previous studies on false memories, we chose not to induce a stereotype threat but to assess instead the older adults’ perceived stereotype threat (PST) when performing such a DRM task. This allowed us to be closer to natural situations of memory examination that older adults encounter (e.g., Rahhal et al., 2001; Brubaker and Naveh-Benjamin, 2018). Besides, since the age-based stereotype threat is known to be higher under difficult tasks (e.g., Steele and Aronson, 1995), we decided to present adults aged 60–70 years with a DRM task associated with a free recall task. First, we expected older adults to correctly recall fewer words as the perception of stereotype threat increases. We also expected the relationship between participants’ perception of stereotype threat and correct recalls to increase with the participants’ level of education. Second, regarding false memories, we expected older adults to produce more critical lures as their perception of a stereotype threat increases. In line with Smith et al. (2017), we expected this relationship to be moderated by education. The relationship between participants’ perception of a stereotype threat and the production of critical lures should be stronger as education increases.

## Materials and Methods

### Participants

Eighty-two older adults (60–70 years, *M* = 64.65 years, *SD* = 3.15, 54% female) were recruited in senior centers and community dwelling homes in the area of Nantes. Participants were all native French speakers; they received no compensation for their participation. All participants gave their informed consent before their inclusion in the study. Global cognitive efficiency was assessed using the French version of the Mini Mental State Examination (MMSE; Derouesné et al., 1999). All older adults scored equal or higher than 26 on the MMSE; results indicated a preserved global cognitive functioning (*M* = 28.74, *SD* = 1.18) in our participants. The French version of the State-Trait Anxiety Inventory (STAI; Spielberger et al., 1993) revealed no signs of trait-anxiety in our sample (*MD_State_* = 29.84, *SD_State_* = 8.62; *M_Trait_* = 37.09, *SD_Trait_* = 8.56). Participants also completed a demographic questionnaire comprising questions regarding the highest diploma they got, the age until which they have been to school, and their current or former occupation. These items were used to compute the Education variable, which is defined as the number of years of instruction since the age of 6 (as instruction was mandatory from the age of 6 in France at the time). Overall, our participants reported a mean of 12.72 years of education (*SD* = 3.12). Participant characteristics are presented in Table 1.

**Table 1:** Participant characteristics: means, SD, and Pearson correlations with confidence intervals.

Variable	*n*	*M*	*SD*	1	2	3	4	5	6	7
1. Age (in years)	82	64.65	3.15							
2. Education (in years)	81	12.72	3.12	−0.34^**^						
				[−0.52, −0.13]						
3. MMSE	82	28.74	1.18	−0.17	0.35^**^					
				[−0.38, 0.05]	[0.14, 0.53]					
4. PST	82	3.54	1.61	0.18	−0.10	−0.16				
				[−0.03, 0.39]	[−0.31, 0.12]	[−0.36, 0.06]				
5. State-anxiety	82	29.84	8.62	0.08	−0.14	−0.09	0.05			
				[−0.14, 0.29]	[−0.35, 0.08]	[−0.30, 0.13]	[−0.17, 0.27]			
6. Trait-anxiety	82	37.09	8.56	0.02	−0.11	0.04	0.14	0.42^**^		
				[−0.20, 0.23]	[−0.32, 0.12]	[−0.18, 0.25]	[−0.08, 0.34]	[0.22, 0.58]		
7. Correct recalls^a^	82	0.53	0.12	−0.32^**^	0.37^**^	0.56^**^	−0.21	−0.10	0.07	
				[−0.50, −0.11]	[0.16, 0.54]	[0.39, 0.69]	[−0.41, 0.01]	[−0.31, 0.12]	[−0.15, 0.28]	
8. Critical lures^a^	82	0.56	0.25	0.13	−0.08	0.00	0.06	−0.14	−0.01	−0.03
				[−0.09, 0.33]	[−0.29, 0.15]	[−0.22, 0.22]	[−0.16, 0.27]	[−0.34, 0.08]	[−0.23, 0.20]	[−0.24, 0.19]

MMSE, Mini Mental State Exam; PST, perceived stereotype threat. Values in square brackets indicate the 95% confidence interval for each correlation.

a Correct recalls and critical lures are reported as proportions.

** *p* < 0.01.

An ethics approval was not required for this study as per institutional and national guidelines and regulations. However, the study was carried out in accordance with University of Nantes ethics guidelines and the French law no. 2004-801 of August 6, 2004 relating to the protection of the natural persons with regard to the processing of personal data and amending law no. 78-17 of January 6, 1978 relating to data, files, and freedoms. Ethics was checked at the laboratory level. Participation in the research was voluntary, and the data were collected anonymously in accordance with the Declaration of Helsinki (World Medical Association, 2013). Participants were informed that they could receive a written document explaining the main results of the study upon request.

### Materials

#### Perception of Stereotype Threat

Participants’ stereotype threat perception was assessed with two items drawn from Gaillard et al. (2011). Participants rated their level of agreement with each of the two statements (“I worry that my ability to perform well on this test is affected by my age” and “I worry that if I perform poorly on this test, the experimenter will attribute my poor performance to my age”) using a 7-point scale ranging from “1-strongly disagree” to “7-strongly agree.” As responses to these two statements were highly correlated (*r* = 0.66, *p* < 0.001), we computed one averaged score of PST.

#### DRM Task

The material used in the present study consisted of six DRM lists, each comprising 15 words, drawn from Corson and Verrier (2007). We created two orders of presentation of the six DRM lists and counterbalanced them across participants.

Descriptive statistics are presented in Table 1.

### Procedure

Participants were tested individually in a single session that lasted approximately 40 min. After giving their informed consent, participants completed the MMSE, the demographic questionnaire, the state-anxiety questionnaire, and then the DRM task. The DRM lists were presented orally by the experimenter, one at a time, at a rate of one word every 1.5 s. Immediately after each list presentation, participants were given 90 s maximum to perform an oral free recall task. Participants were told that the experimenter was more interested in the type of words (quality of the recall) than in the number of words they recall (quantity of the recall). Immediately after the DRM task, participants assessed their perception of stereotype threat and then their level of trait-anxiety. At the end of the session, participants were fully debriefed and thanked for their participation.

## Results

The results are presented in the next three sections. We first examined the correlations between all the variables taken into account in our study. Second, we examined the moderator role of education on the relationship between PST and the correct recalls. Finally, we investigated the moderating role of education on the relationship between PST and false memories production (production of critical lures). Statistical analyses were performed using R software (Version 3.6.0; R Core Team, 2019) and JASP (Version 0.13.1; JASP Team, 2020).

### Correlation Analyses

The analysis revealed that Education was negatively and significantly correlated with Age but positively and significantly correlated with global cognitive efficiency assessed with the MMSE. Higher levels of education were associated with higher global cognitive efficiency and younger age. As expected, the two measures of state‐ and trait-anxiety were positively and significantly correlated. In addition, proportions of correct recalls in the DRM task were significantly and positively correlated with Education and global cognitive efficiency but negatively with Age. No other significant correlation was found (see Table 1).

### Correct Recalls

We conducted a regression analysis to investigate both the role of PST as a predictor of the proportion of correct recalls and the moderating role of Education on the proportions of correct recalls in the DRM task (see Table 2).

**Table 2:** Hierarchical regression analyses for variables predicting the proportion of correct recalls.

Predictor	*b*	*p*	*b* 95% CI	*sr* ^2^	*sr* ^2^ 95% CI	Fit	Difference
Model 1							
(Intercept)	0.53	<0.001	[0.50, 0.61]				
Education	0.01	0.001	[−0.03, 0.04]	0.00	[−0.02, 0.02]		
PST	−0.01	0.155	[−0.02, 0.01]	0.00	[−0.03, 0.04]		
						*R* ^2^ = 0.156 [0.03, 0.06]	
						*p* = 0.738	
Model 2							
(Intercept)	0.54	< 0.001	[0.51, 0.56]				
Education	0.01	0.002	[0.00, 0.02]	0.11	[−0.01, 0.23]		
PST	−0.01	0.235	[−0.03, 0.01]	0.02	[−0.03, 0.06]		
PST × Education	0.01	0.068	[−0.00, 0.01]	0.04	[−0.04, 0.11]		
						*R* ^2^ = 0.192 [0.04, 0.32]	Δ*R* ^2^ = 0.036 [0.00, 0.13]
						*p* < 0.001	*p* = 0.068
Model 3							
(Intercept)	−0.30	0.447	[−1.09, 0.49]				
Education	0.01	0.176	[−0.00, 0.01]	0.02	[−0.03, 0.06]		
PST	−0.01	0.442	[−0.02, 0.01]	0.00	[−0.02, 0.03]		
PST × Education	0.00	0.306	[−0.00, 0.01]	0.01	[−0.02, 0.04]		
Age	−0.01	0.059	[−0.01, 0.00]	0.03	[−0.03, 0.09]		
STAI.State	−0.00	0.655	[−0.00, 0.00]	0.00	[−0.01, 0.02]		
STAI.Trait	0.00	0.278	[−0.00, 0.00]	0.01	[−0.02, 0.04]		
MMSE	0.04	< 0.001	[0.02, 0.06]	0.15	[0.02, 0.27]		
						*R* ^2^ = 0.401 [0.17, 0.49]	Δ*R* ^2^ = 0.209 [0.07, 0.35]
						*p* < 0.001	*p* < 0.001

PST, perceived stereotype threat. A significant *b*-weight indicates that the semi-partial correlation is also significant. *b* represents unstandardized regression weights. *sr* ^2^ represents the semi-partial correlation squared.

First, PST and Education were introduced as predictors of the proportion of correct recalls (Model 1). The analysis yielded a significant main effect of Education (*b* = 0.01, *p* = 0.001, *sr* ^2^ = 0.00). When the interaction between Education and PST was added to the model, the results indicated that 19% of the variance of the correct recalls is related to the model (*p* < 0.001). More precisely, Education was the only significant predictor of the proportion of correct recalls (*b* = 0.01, *p* = 0.002, *sr* ^2^ = 0.11). Neither the PST (*b* = −0.01, *p* = 0.235) nor the Education × PST interaction (*b* = 0.01, *p* = 0.068) predicted the proportion of correct recalls (Model 2).

Finally, we added Age, MMSE, State-, and Trait-Anxiety to the previous model as controlled variables (Model 3). The overall fit increases significatively (*R* ^2^ = 0.401, *p* < 0.001; Δ*R* ^2^ = 0.209, *p* < 0.001) and MMSE became the only significant predictor (*b* = 0.04, *p* < 0.001, *sr* ^2^ = 0.15) offsetting the effect of Education (*b* = 0.01, *p* = 0.176, *sr* ^2^ = 0.02).

### False Memories

We conducted a regression analysis to investigate both the role of PST as a predictor of the proportion of critical lures produced and the moderating role of Education on the proportions of critical lures produced in the DRM task (see Table 3).

**Table 3:** Hierarchical regression analyses for variables predicting the proportion of critical lures.

Predictor	*b*	*p*	*b* 95% CI	*sr* ^2^	*sr* ^2^ 95% CI	Fit	Difference
Model 1							
(Intercept)	0.56	<0.001	[0.50, 0.61]				
PST	0.01	0.691	[−0.03, 0.04]	0.00	[−0.02, 0.02]		
Education	−0.01	0.531	[−0.02, 0.01]	0.00	[−0.03, 0.04]		
						*R* ^2^ = 0.008 [0.00, 0.06]	
						*p* = 0.738	
Model 2							
(Intercept)	0.56	<0.001	[0.51, 0.62]				
PST	0.01	0.455	[−0.02, 0.05]	0.01	[−0.03, 0.04]		
Education	−0.01	0.364	[−0.03, 0.01]	0.01	[−0.03, 0.05]		
PST × Education	0.01	0.013	[0.00, 0.03]	0.08	[−0.03, 0.19]		
						*R* ^2^ = 0.084 [0.00, 0.19]	Δ*R* ^2^ = 0.076 [0.00, 0.22]
						*p* = 0.078	*p* = 0.013
Model 3							
(Intercept)	0.17	0.858	[−1.76, 2.11]				
PST	0.01	0.593	[−0.03, 0.04]	0.00	[−0.02, 0.03]		
Education	−0.01	0.559	[−0.03, 0.01]	0.00	[−0.02, 0.03]		
PST × Education	0.01	0.020	[0.00, 0.03]	0.07	[−0.04, 0.17]		
Age	0.01	0.315	[−0.01, 0.03]	0.01	[−0.03, 0.06]		
STAI.State	−0.00	0.234	[−0.01, 0.00]	0.02	[−0.04, 0.07]		
STAI.Trait	0.00	0.906	[−0.01, 0.01]	0.00	[−0.01, 0.01]		
MMSE	−0.00	0.882	[−0.05, 0.05]	0.00	[−0.01, 0.01]		
						*R* ^2^ = 0.115 [0.00, 0.18]	Δ*R* ^2^ = 0.031[−0.04, 0.10]
						*p* = 0.238	*p* = 0.638

PST, perceived stereotype threat. A significant *b*-weight indicates that the semi-partial correlation is also significant. *b* represents unstandardized regression weights. *sr* ^2^ represents the semi-partial correlation squared.

First, PST and Education were introduced as predictors of the proportion of critical lures. The analysis yielded no significant main effect (Model 1). When the interaction between Education and PST was introduced, the results indicated that the model predicted 8% of the total variance (*p* = 0.078). More precisely, neither the PST (*b* = 0.01, *p* = 0.455, *sr* ^2^ = 0.01) nor the Education (*b* = −0.01, *p* = 0.364, *sr* ^2^ = 0.01) predicted the proportion of critical lures (Model 2). However, results indicated a moderating role of Education with 8% of the total variance associated with the PST × Education interaction (*b* = 0.01, *p* = 0.013, *sr* ^2^ = 0.08); the relation between PST and the production of critical lures depends on an individual’s education level.

When Age, MMSE, State-, and Trait-Anxiety were entered in the previous model as controlled variables, the overall fit of the model increases nonsignificantly (*R* ^2^ = 0.11, *p* = 0.238; Δ*R* ^2^ = 0.031, *p* = 0.638), but PST × Education interaction still was the only significant predictor (*b* = 0.01, *p* = 0.020, *sr* ^2^ = 0.07) confirming the moderating role of Education on the relationship between PST and the production of critical lures (Model 3).

**Figure 1:**
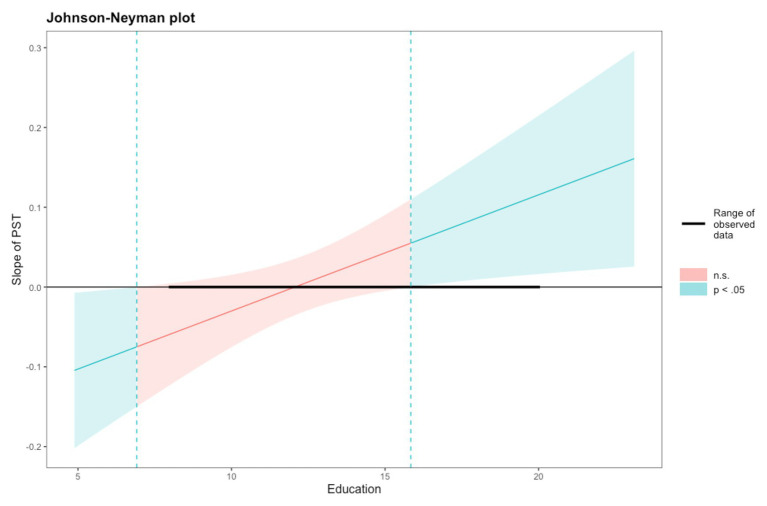
Representation of the slope of perceived stereotype threat on the production of critical lures according to the level of education. The range of observed values of Education is [8.00, 20.00].

One way to visualize and summarize simply the moderated effect of Education is to follow the Johnson-Neyman technique (Johnson and Fay, 1950). From Model 2 in Table 3, we used this technique to determine the education level intervals in which the relationship between PST and the proportion of critical lures is significant or not significant (see Figure 1). When the level of education is between 6.9 and 15.8 years, the relationship between PST and the proportion of critical lures is not significant; when the level is above 15.8 years, an increase in PST is significantly related to an increase in the proportion of critical lures, and when the level is below 6.9 years, an increase in PST is significantly related to a decrease in the proportion of critical lures. For instance, for the higher educated group (>16 years), the slope of PST on the proportion of critical lures is greater than *b* = 0.06 (and significant). One should note, however, that lower levels of Education such as 6.9 years and less are scarce in adults now aged from 60 and over.

## Discussion

The present study was aimed at identifying the moderating role of education on the relationship between the perceived age-based stereotype threat and false memory. As expected, our results showed that the production of critical lures was best predicted by the PST × Education interaction. To our knowledge, this is the first study that shows an increase of false memories’ production in highly educated older adults as their perception of an age-based stereotype threat increases, without relying on any experimental stereotype threat manipulation. Our results are consistent with previous research that has shown that highly educated older adults are more susceptible to false memories (Smith et al., 2017) under the induced stereotype threat. These results support the idea that highly educated individuals may place a high value on their memory and feel especially threatened by age-based stereotypes in such a false memory task. Consistent with the cognitive hypothesis (Schmader and Johns, 2003), they may engage most of their cognitive resources in order to distance themselves from the stereotype, thereby leaving only few resources to correctly perform the task. The increase of false memories in these highly educated participants along with the increase of their perception of an age-based stereotype threat is also consistent with the increase of false alarms observed by Brubaker and Naveh-Benjamin (2018) in adults aged 65 and over under the stereotype threat and the increase of false memories showed by Thomas and Dubois (2011) and Smith et al. (2017). This is particularly interesting since, in contrast to most previous studies, we did not use experimental stereotype threat induction. This suggests that naturally occurring stereotype threat in testing situations (e.g., Brubaker and Naveh-Benjamin, 2018) may have as deleterious effects on cognitive performance as an induced stereotype threat.

The literature shows that the occurrence of false memories is usually the result of global, heuristic, relational, familiarity-based, or gist-based processing rather than in distinctive, item-specific, or verbatim processing (e.g., Smith and Hunt, 1998; Brainerd and Reyna, 2002; McCabe et al., 2004). Our results indicate that participants with a strong PST may have engaged in a familiarity-based or gist-based strategy rather than in a recollection or verbatim processing during the free recall task. This higher reliance on familiarity leads participants to falsely recall critical lures. This is consistent with the idea that under threat, participants may rely more on global, heuristic, or automatic processes than on analytic and controlled processes (Mazerolle et al., 2012). This strategy is quite maladaptive in the false memory context but is particularly effective when trying to recall studied words from DRM lists. Indeed, with regards to correct recalls, our results showed that education is the only predictor of participants’ performance; the more educated they are, the more they recall previously studied words. This positive effect of education is consistent with previous research showing that education, as a major component of cognitive reserve, is a protective factor against the decline of cognitive abilities in aging (e.g., Nyberg and Pudas, 2019; Clouston et al., 2020). However, when MMSE total score was controlled for, it became the only significant predictor of correct recalls, offsetting the effect of education. In addition, we showed a strong positive correlation between MMSE total score and the proportion of correct recalls. These results are consistent with previous studies that have highlighted the positive relationship between MMSE total score and episodic memory performance (e.g., Taylor et al., 1992; Aartsen et al., 2002). Our results also pointed to a positive correlation between MMSE total score and education. This is consistent with previous studies showing that higher levels of education are associated with better cognitive functioning (e.g., Crum, 1993; Albert et al., 1995; but see Bleecker et al., 1988) or that education is a good predictor of the MMSE total score (e.g., Bertolucci et al., 1994). Finally, we highlighted a significant negative correlation between age and education level which is consistent with previous studies showing that earlier born cohorts of adults received less education than those born later (e.g., Matthews et al., 2012).

Contrary to our expectations and to previous research, our results did not show any effect of a perceived of stereotype threat either on correct recalls (e.g., Hess et al., 2009; Barber and Mather, 2013b; Armstrong et al., 2017) or on false memories (Thomas and Dubois, 2011; Smith et al., 2017). One explanation might be that, in contrast with previous studies, we did not use a blatant stereotype threat induction using short texts describing cognitive and memory declines in aging. To be closer to natural situations of memory assessments, we chose to assess participants’ PST naturally occurring in a testing situation. Our participants reported scores of PST as high as those reported by the participants in Gaillard et al. (2011) who underwent an experimental stereotype threat induction. As participants were informed both in the consent form and in the instructions that the experiment was about memory, one cannot rule out the possibility that this constitutes a subtle induction of stereotype threat (Nguyen and Ryan, 2008; Marquet et al., 2016). Another explanation may lie in the fact that we used a DRM associated with a free recall task that, despite our specific instructions regarding the recall task, may have elicited a promotion focus in our participants. Since it has been argued that the stereotype threat induces a prevention focus (see Barber, 2017), the lack of regulatory fit may have led to these results. However, our study did not directly address the regulatory focus theory since we did not manipulate nor assess promotion or prevention foci in our participants. Future research using subtle induction of stereotype threat would benefit from investigating this issue further.

In conclusion, this study showed that an age-based stereotype threat may naturally occur under testing situations in adults aged between 60 and 70 and affect their susceptibility to false memories. The present study also highlighted the central role of education in the evaluation of memory in older adults. While it predicted correct recalls, it also moderated the effects of PST on false memories. These results encourage more consideration of the education level as a study variable in research on aging, and not just as a simple controlled variable.

## Data Availability Statement

The raw data supporting the conclusions of this article will be made available by the authors, without undue reservation.

## Ethics Statement

Ethical review and approval was not required for the study on human participants in accordance with the local legislation and institutional requirements. The patients/participants provided their written informed consent to participate in this study.

## Author Contributions

A-LG, CE, and FC contributed to conception and design of the study. J-MG conducted the statistical analyses. A-LG wrote the first draft of the manuscript. CE, FC, and J-MG provided comments on the manuscript. All authors contributed to manuscript revision and read and approved the submitted version.

### Conflict of Interest

The authors declare that the research was conducted in the absence of any commercial or financial relationships that could be construed as a potential conflict of interest.
